# Digital oximetry biomarkers for assessing respiratory function: standards of measurement, physiological interpretation, and clinical use

**DOI:** 10.1038/s41746-020-00373-5

**Published:** 2021-01-04

**Authors:** Jeremy Levy, Daniel Álvarez, Aviv A. Rosenberg, Alexandra Alexandrovich, Félix del Campo, Joachim A. Behar

**Affiliations:** 1 Faculty of Biomedical Engineering, Technion Institute of Technology, Haifa, Israel; 2 Faculty of Electrical Engineering, Technion Institute of Technology, Haifa, Israel; 3 Río Hortega University Hospital Valladolid, Valladolid, Spain; 4 Biomedical Engineering Group, University of Valladolid, Valladolid, Spain; 5 Centro de Investigación Biomédica en Red en Bioingeniería, Biomateriales y Nanomedicina (CIBER-BBN), Valladolid, Spain; 6 Department of Computer Science, Technion Institute of Technology, Haifa, Israel

**Keywords:** Diagnostic markers, Prognostic markers, Predictive markers

## Abstract

Pulse oximetry is routinely used to non-invasively monitor oxygen saturation levels. A low oxygen level in the blood means low oxygen in the tissues, which can ultimately lead to organ failure. Yet, contrary to heart rate variability measures, a field which has seen the development of stable standards and advanced toolboxes and software, no such standards and open tools exist for continuous oxygen saturation time series variability analysis. The primary objective of this research was to identify, implement and validate key digital oximetry biomarkers (OBMs) for the purpose of creating a standard and associated reference toolbox for continuous oximetry time series analysis. We review the sleep medicine literature to identify clinically relevant OBMs. We implement these biomarkers and demonstrate their clinical value within the context of obstructive sleep apnea (OSA) diagnosis on a total of *n* = 3806 individual polysomnography recordings totaling 26,686 h of continuous data. A total of 44 digital oximetry biomarkers were implemented. Reference ranges for each biomarker are provided for individuals with mild, moderate, and severe OSA and for non-OSA recordings. Linear regression analysis between biomarkers and the apnea hypopnea index (AHI) showed a high correlation, which reached $\overline R ^2 = 0.82$. The resulting python OBM toolbox, denoted “pobm”, was contributed to the open software PhysioZoo (physiozoo.org). Studying the variability of the continuous oxygen saturation time series using pbom may provide information on the underlying physiological control systems and enhance our understanding of the manifestations and etiology of diseases, with emphasis on respiratory diseases.

## Introduction

Pulse oximetry is routinely used for non-invasive monitoring of oxygen saturation levels. A low oxygen level in the blood means low oxygen in the tissues, which can ultimately lead to organ failure. Oximetry can be used to sporadically measure the oxygen saturation level during a medical examination or continuously monitor patients in the intensive care unit (ICU) or overnight for a polysomnography (PSG) study. Identification of digital biomarkers extrapolated from the oxygen saturation time series can support the diagnosis and continuous monitoring of patient pulmonary function to predict deteriorations (prognosis). Specifically, studying the variability of the oxygen saturation signal may provide information on the underlying physiological control systems. Furthermore, it may enhance our understanding of the manifestation and etiology of diseases and identify digital oximetry biomarkers (OBMs) for the purpose of health monitoring. Sleep medicine makes standard usage of oximetry biomarkers, where overnight drops in oxygen saturation are characteristic of obstructive sleep apnea (OSA). Beyond the presence of OSA, the repetitive nocturnal hypoxemia may cause oxidative stress, contributing to the pathogenesis of cardiovascular morbidity^1^. Similarly, patients with advanced chronic obstructive pulmonary disease (COPD), and with no primary sleep-related breathing disorders, commonly exhibit overnight hypoxemia^2^. Yet, contrary to heart rate variability (HRV) measures, a field which has benefited from the development of stable standards^3^ and advanced toolboxes and software^4–6^, there are currently no such standards and open tools for analyzing oxygen saturation time series in terms of its variability, dynamics, and the statistical characterization of specific patterns.

### This contribution

Research on the use of existing and development of new oximetry biomarkers has mainly focused on the diagnosis of OSA, as echoed by five recent reviews in this field^7–11^. Although this paper will naturally somewhat overlap with these reviews, we present a new comprehensive review focusing on the physiological interpretation and clinical use of oximetry biomarkers, in the spirit of the work of Malik et al.^3^ in the field of HRV analysis. We also develop a complete Python toolbox (denoted “pobm”) and software interface (“PhysioZoo OBM”) for usage of these biomarkers, similar to our previous work in HRV analysis^6^. This will support rigorous research in oximetry time series analysis and ensure reproducibility of research. We apply the developed toolbox to a large dataset of overnight recordings in order to demonstrate its usability and clinical value in the context of OSA diagnosis. While the work mainly focuses on OBM developed in the field of sleep medicine, the reviewed OBMs can be applied to the analysis of continuous oximetry recordings for any other condition and we thus introduce a general purpose flow diagram for continuous oximetry analysis. We limit the scope of the biomarkers to single-channel oximetry analysis, thereby implicitly excluding OBMs that may require additional channels, such as airflow, to be engineered^12^. The paper defines categories of OBMs and a review of the literature to identify evidence-based OBMs in the field of sleep medicine as well as some additional suggested OBMs. The biomarkers are applied to a large PSG dataset totaling 3806 individual oximetry recordings.

## Results

### pobm toolbox and PhysioZoo OBM interface

**Fig. 1:**
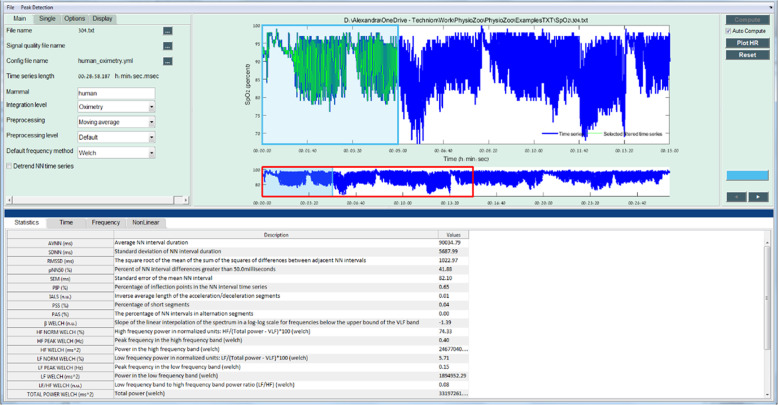
PhysioZoo OBM interface for oximetry time series analysis. The analysis of an oximetry time series is shown. All the OBM biomarkers are computed for the selected window (in light green).

The pobm toolbox was implemented in Python. For the purpose of quality control, functions were benchmarked against comparative reference source code (Supplementary Table 1) or ranges published in the literature (Supplementary Table 2). For the comparison to range reported in the literature, we compared the order of magnitude of some biomarkers with those reported by other for the non-OSA group. Figure 1 shows the PhysioZoo OBM interface for oximetry analysis. In PhysioZoo OBM, a SpO_2_ time series can be loaded (File → Open data file) and pre-filtered using one of the preprocessing filters introduced in section “Preprocessing”. After computation, OBM can be exported together with standard data representation figures. The PhysioZoo software handles data in .txt, .mat (The MathWorks, Inc., Natick, MA, USA) and WFDB^13^ formats. In addition, the PhysioZoo OBM enables oximetry analysis of multiple segments, thereby enabling tracking of temporal changes in oximetry measures for a given record. The pobm toolbox and PhysioZoo software are available at https://physiozoo.com/.

### Standard ranges for oximetry biomarkers

Tables 1 and 2 summarize the median and interquartile range for all the OBMs implemented in the PhysioZoo software for individuals participating in SHHS1. This provides a standard reference range for each oximetry biomarker. The null hypothesis of Kruskal–Wallis test was rejected for most biomarkers (43/44) with the smallest *p* value obtained for Px, CA_*x*_, and CT_*x*_. Following the Dunn post hoc analysis, a total of 30 biomarkers were statistically discriminative between every pair of classes, i.e., *p* < 0.05 for all pairs (Tables 1 and 2).

**Table 1:** Summary statistics (median and interquartiles Q1–Q3) for the General statistics, Complexity and Periodicity OBMs evaluated on the SHHS1 database.

Biomarker	Non-OSA (*n* = 1195)	Mild (*n* = 1303)	Moderate (*n* = 833)	Severe (*n* = 475)	Kruskal test
General statistics					
AV	95.90 (94.77–96.93)	95.14 (94.01–96.09)	94.43 (93.36–95.41)	93.51 (92.23–94.67)	*p* < 0.001^‡^
MED	96.00 (95.00–97.00)	95.00 (94.00–96.00)	95.00 (93.00–96.00)	94.00 (93.00–95.00)	*p* < 0.001^‡^
Min	79.00 (72.00–85.00)	79.00 (74.00–84.00)	79.00 (73.00–83.00)	76.00 (70.00–81.00)	*p* < 0.001
SD	1.42 (1.08–1.90)	1.55 (1.29–1.90)	1.81 (1.50–2.19)	2.33 (1.88–3.04)	*p* < 0.001^‡^
RG	20.00 (14.00–27.00)	19.00 (14.00–25.00)	20.00 (15.00–26.00)	22.00 (18.00–29.00)	*p* < 0.001
Pxx	91.00 (88.00–93.00)	90.00 (88.00–92.00)	89.00 (86.00–90.12)	85.00 (81.00–88.00)	*p* < 0.001^‡^
Mx	2.14 (1.18–3.82)	3.44 (1.94–5.51)	5.84 (3.49–8.96)	10.98 (6.78–17.00)	*p* < 0.001^‡^
ZCxx	435.00 (346.00–540.00)	481.00 (391.00–579.00)	557.00 (463.50–638.00)	690.00 (592.00–812.50)	*p* < 0.001^‡^
ΔI	0.36 (0.30–0.42)	0.47 (0.41–0.54)	0.66 (0.57–0.76)	1.03 (0.86–1.44)	*p* < 0.001^‡^
Complexity					
ApEn	0.17 (0.13–0.20)	0.21 (0.17–0.26)	0.28 (0.23–0.34)	0.39 (0.31–0.48)	*p* < 0.001^‡^
LZ	2389.00 (2241.00–2511.00)	2435.00 (2314.00–2547.00)	2492.00 (2374.50–2587.50)	2587.00 (2483.50–2698.00)	*p* < 0.001^‡^
CTMx	0.92 (0.90–0.93)	0.89 (0.88–0.91)	0.86 (0.84–0.87)	0.79 (0.73–0.82)	*p* < 0.001^‡^
SampEn	0.06 (0.04–0.08)	0.08 (0.06–0.11)	0.12 (0.09–0.15)	0.21 (0.14–0.31)	*p* < 0.001^‡^
DFA	1.14 (0.99–1.37)	1.35 (1.18–1.54)	1.70 (1.49–1.98)	2.47 (2.05–3.24)	*p* < 0.001^‡^
Periodicity					
PRSAD_c_	0.56 (0.53–0.60)	0.56 (0.54–0.59)	0.59 (0.56–0.63)	0.67 (0.61–0.78)	*p* < 0.001^‡^
PRSAD_ad_	1.55 (1.34–1.89)	1.83 (1.58–2.16)	2.42 (2.01–2.97)	3.68 (2.86–4.94)	*p* < 0.001
PRSAD_os_	0.11 (0.09–0.13)	0.13 (0.11–0.15)	0.17 (0.14–0.20)	0.25 (0.20–0.33)	*p* < 0.001^‡^
PRSAD_sb_	0.01 (0.00–0.03)	0.03 (0.02–0.05)	0.07 (0.05–0.10)	0.14 (0.09–0.21)	*p* < 0.001^‡^
PRSAD_sa_	0.02 (0.01–0.04)	0.04 (0.03–0.06)	0.08 (0.05–0.11)	0.14 (0.09–0.20)	*p* < 0.001
AC(x10^4^)	18.20 (17.83–18.54)	17.97 (17.58–18.29)	17.69 (17.30–18.04)	17.33 (16.90–17.79)	*p* < 0.001
PSD_total	91.49 (89.48–93.71)	94.65 (92.36–97.49)	99.81 (96.76–103.93)	109.27 (103.81–118.61)	*p* < 0.001^‡^
PSD_band	3.22 (2.78–3.91)	4.28 (3.66–5.06)	6.18 (5.18–7.39)	9.46 (7.60–13.42)	*p* < 0.001^‡^
PSD_ratio	0.04 (0.03–0.04)	0.05 (0.04–0.05)	0.06 (0.05–0.07)	0.09 (0.07–0.11)	*p* < 0.001^‡^
PSD_peak	0.02 (0.02–0.02)	0.03 (0.02–0.03)	0.04 (0.03–0.05)	0.06 (0.05–0.10)	*p* < 0.001^‡^

The symbol ‡ indicate when the result of the Dunn post hoc test between all pairs of groups was *p* < 0.05. Results presented for the categories general statistics, complexity, and periodicity.

**Table 2:** Summary statistics (median and interquartiles Q1–Q3) for the Desaturations and Hypoxic Burden OBMs evaluated on the SHHS1 database.

Biomarker	Non-OSA (*n* = 1195)	Mild (*n* = 1303)	Moderate (*n* = 833)	Severe (*n* = 475)	Kruskal test
Desaturations					
DL_*μ*_	39.44 (34.38 to 45.23)	41.95 (37.00 to 47.31)	42.10 (37.24 to 46.98)	38.04 (33.30 to 43.11)	*p* < 0.001
DL_*σ*_	23.04 (18.65 to 26.82)	22.94 (19.62 to 26.16)	21.68 (18.69 to 24.36)	18.10 (15.10 to 21.04)	*p* < 0.001
DL_*σ*_	4.93 (4.11 to 6.06)	4.60 (4.12 to 5.27)	4.53 (4.13 to 5.16)	5.00 (4.39 to 6.02)	*p* < 0.001^‡^
DDmax_*σ*_	2.63 (1.43 to 3.97)	2.24 (1.48 to 3.26)	2.20 (1.57 to 2.95)	2.48 (1.84 to 3.34)	*p* < 0.001
DD100_*μ*_	9.47 (8.20 to 10.91)	9.38 (8.42 to 10.50)	9.57 (8.54 to 10.63)	10.28 (8.98 to 11.88)	*p* < 0.001
DD100^*σ*^	3.24 (1.85 to 5.06)	2.78 (1.90 to 4.03)	2.60 (1.95 to 3.58)	2.74 (2.11 to 3.84)	*p* < 0.001
DS_*μ*_	−0.22 (−0.26 to 0.18)	−0.19 (−0.23 to 0.16)	−0.18 (−0.21 to 0.16)	−0.20 (−0.25 to 0.17)	*p* < 0.001^‡^
DS_*σ*_	0.14 (0.11 to 0.19)	0.13 (0.10 to 0.16)	0.11 (0.09 to 0.14)	0.11 (0.09 to 0.14)	*p* < 0.001^‡^
DAmax_*μ*_	104.74 (80.48 to 137.64)	104.55 (87.47 to 126.95)	108.16 (90.44 to 130.04)	115.22 (96.08 to 144.56)	*p* < 0.001
DAmax_*σ*_	87.52 (53.67 to 144.54)	82.72 (58.97 to 122.55)	85.36 (64.67 to 117.12)	94.62 (70.51 to 127.90)	*p* < 0.001
DA100_*μ*_	280.75 (223.36 to 352.90)	304.38 (251.74 to 362.10)	315.47 (258.35 to 377.54)	313.76 (262.60 to 380.12)	*p* < 0.001
DA100_*σ*_	169.66 (120.29 to 262.66)	175.29 (133.78 to 233.17)	177.53 (134.72 to 222.65)	175.48 (135.08 to 225.74)	*p* > 0.05
TD_*μ*_	1760.82 (1122.78 to 2804.90)	940.56 (673.65 to 1373.38)	429.00 (321.28 to 617.51)	181.61 (117.70 to 268.42)	*p* < 0.001^‡^
TD_*σ*_	2365.50 (1660.51 to 3388.55)	1457.2 (1092.66 to 2014.68)	812.64 (635.57 to 1066.14)	375.70 (222.57 to 544.07)	*p* < 0.001
Hypoxic burden					
POD_*x*_	1.72 (1.69 to 01.76)	10.20 (9.60 to 11.00)	18.23 (18.11 to 18.79)	31.10 (30.02 to 33.65)	*p* < 0.001^‡^
AODmax	0.06 (0.03 to 0.10)	0.11 (0.07 to 0.16)	0.24 (0.16 to 0.36)	0.61 (0.37 to 1.05)	*p* < 0.001^‡^
AOD100	0.15 (0.09 to 0.26)	0.31 (0.20 to 0.47)	0.70 (0.46 to 1.00)	1.65 (1.04 to 2.77)	*p* < 0.001^‡^
CT_*x*_	0.51 (0.15 to 1.34)	0.76 (0.30 to 1.69)	1.42 (0.58 to 3.29)	4.48 (1.68 to 11.58)	*p* < 0.001^‡^
CA_*x*_	0.45 (0.13 to 1.10)	0.64 (0.25 to 1.42)	1.21 (0.51 to 2.79)	3.84 (1.42 to 9.86)	*p* < 0.001^‡^

The symbol ‡ indicate when the result of the Dunn post hoc test between all pairs of groups was *p* < 0.05. Results presented for the categories desaturation measures and hypoxic burden.

### Added value in combining multiple biomarkers

**Fig. 2:**
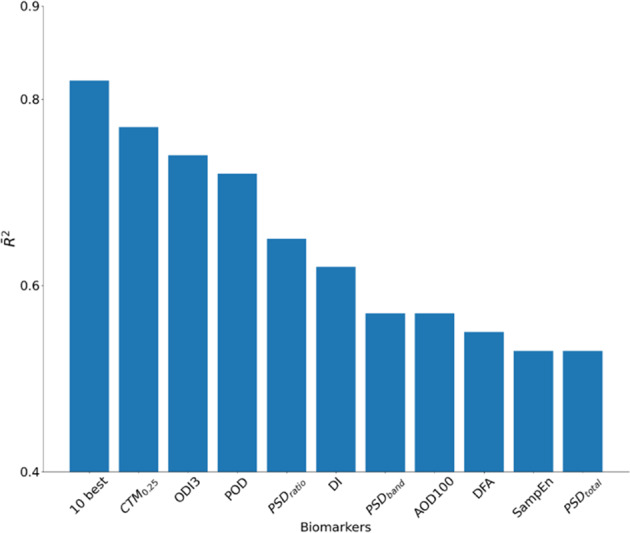
Linear correlation between oximetry biomarkers and the AHI. The adjusted $\overline R ^2$ is reported for every biomarker and for the 10 biomarkers with the highest $\overline R ^2$.

When performing simple regression analysis for individual OBM against the AHI, CTM_*ρ*_ with *ρ* = 0.25 achieved the highest goodness of fit ($\overline R ^2 = 0.77$) followed by the ODI3 ($\overline R ^2 = 0.74$). When combining the 10 oximetry biomarkers with the best score within a multivariable linear regression framework, the goodness of fit was further improved to $\overline R ^2 = 0.82$ (Fig. 2).

## Discussion

We showed that OBMs engineered from continuous oximetry recordings may provide discriminative information of groups of individuals suffering from respiratory disorders. Within the context of OSA, we found that CTM_*ρ*_ with *ρ* = 0.25 had the highest $\overline R ^2$ in estimating the AHI, with $\overline R ^2 = 0.77$. Furthermore, we demonstrated that combining multiple oximetry biomarkers for estimating the AHI increased the $\overline R ^2$ to 0.82. This highlights the complementary value in using multiple OBMs versus a single one.

Recent studies have shown that nocturnal hypoxemia correlates better with cardiovascular disease, cancer incidence, and mortality than traditional nocturnal respiratory disturbance indexes, such as the AHI^12,14^. This suggests that alternative nocturnal OBMs may provide important health information. Both intermittent hypoxia and sleep fragmentation are responsible for clinical manifestations and most related comorbidities of OSA^15^. In OSA, recurrent collapse of the upper airway leads to a reduced tidal volume and both intermittent hypoxemia and hypercapnia. In consequence, activity of the sympathetic nervous system increases and cortical arousals occur, leading to disrupted sleep architecture and restless sleep. In addition, repetitive hypoxemia–reoxygenation periods are linked to the production of free oxygen radicals, inflammation, and endothelial dysfunction^16^. In this regard, OBMs, such as CT90 and overnight mean and minimum saturation, have been significantly linked with dysfunction in cardiovascular modulation, arterial hypertension, atrial fibrillation, increased insulin resistance, higher incidence of lung cancer, and worst prognosis after myocardial infarction, as well as higher risk of post-surgery complications in OSA patients^17^. However, it remains very unclear which oximetric biomarkers (or combinations of biomarkers) are most predictive of clinical endpoints, such as metabolic and cardiovascular diseases. For example, the average duration and morphology of the events are not considered in routine OSA diagnosis. This is limiting, since longer apnea or hypopnea events will likely result in increased desaturation (in length and depth), which will likely result in more hypoxic stress, leading to more severe cardiovascular consequences. At the same time, longer desaturation events may result in a decrease in AHI, that is, a lower number of events per hour. Thus the relationship between duration and morphology of events and a clinical endpoint (e.g., cardiovascular complication) remains unclear. For this reason, additional desaturation biomarkers may provide valuable information on disease phenotyping. This has been suggested, for example, in the work of Kulkas et al.^18^, who showed, albeit on a very small population sample (*n* = 19), that additional oximetry biomarkers, i.e., duration and morphology related, enhance OSA phenotyping.

Nocturnal hypoxemia can be present in many respiratory diseases that are either acute or chronic. For example, OSA patients show cyclic desaturation–resaturation episodes during the night, which are linked with partial or complete obstruction of the upper airway, leading to the well-known chronic intermittent hypoxia pattern. On the other hand, COPD patients show slower and longer desaturations linked with sustained hypoventilation, mainly during rapid eye movement (REM) sleep, leading to a state of nocturnal chronic hypoxemia. Characterization of hypoxemia is therefore different during REM sleep. In subjects with OSA, oxygen desaturation indices (ODIs) of 3 and 4%, as well as mean, minimum, and CT90, are widely used in sleep medicine. On the contrary, criteria for classifying a COPD patient as a nocturnal desaturator are not well established. Showing at least one episode with saturation <90% lasting for >5 min and reaching a minimum saturation of at least 85% has been proposed^19^, while some authors define nocturnal desaturators as patients with CT90 ≥30%^20^. This example highlights that a standard to characterize and quantify some respiratory conditions, such as COPD, using nocturnal oximetry, remain to be defined. Usage of multiple OBM may also enable to identify patterns for different apnea types such as central apnea versus mixed apnea versus obstructive apnea as ongoing research studies such as the SomnaPatch intend (Somnarus Inc., ClinicalTrials.gov Identifier: NCT02034175). Machine learning algorithms will play an important role in engineering models that can learn complex combinations of OBM for the purpose of regression or classification tasks. Such models will uncover the OBM combinations that best reflect the unique patterns of a given condition.

**Fig. 3:**
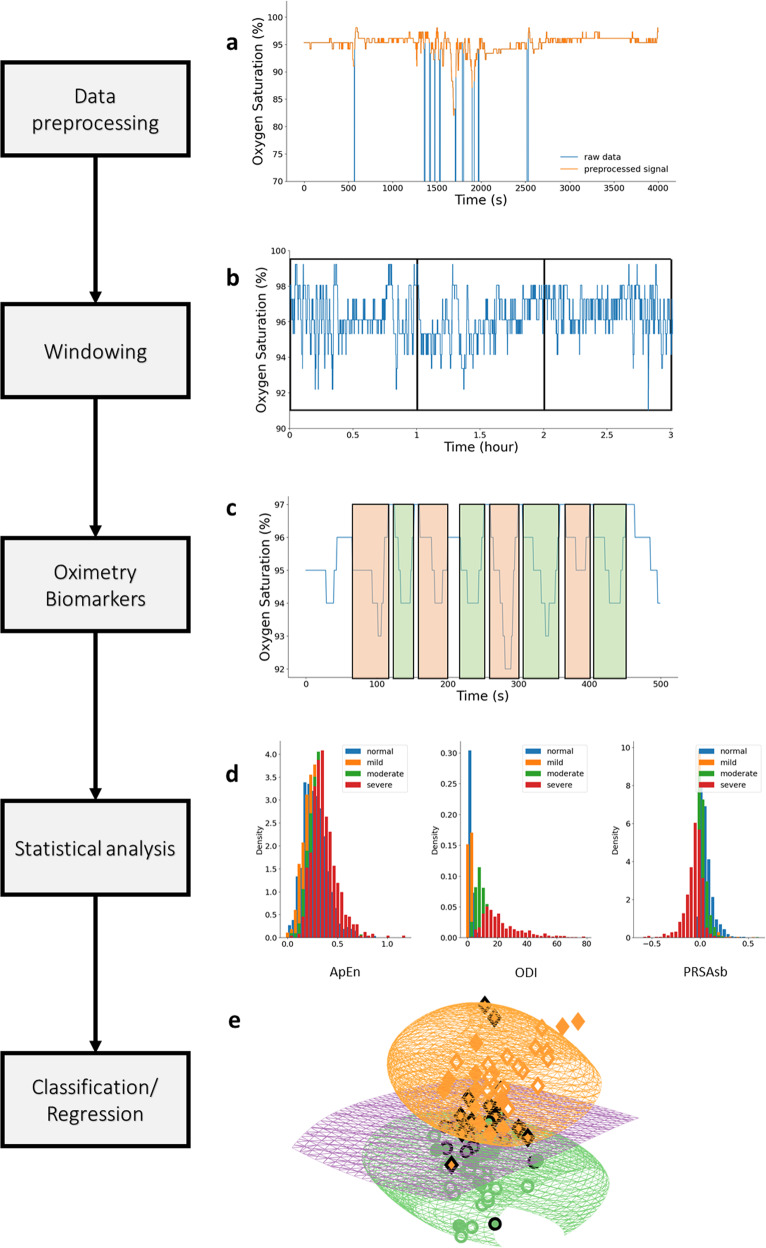
Flowchart for continuous oximetry time series analysis. **a** The raw data are first preprocessed using some of the routines presented in section “Preprocessing”, to remove any non-physiological values that are likely caused by noise. **b** The recording is then windowed. The size of the analysis windows and whether to consider overlapping windowing is an important consideration and will be research dependent. **c** Oximetry biomarkers are then computed using the OBM toolbox. **d** A statistical analysis is performed to obtain a preliminary understanding of the biomarker behavior for the different classes of interest. **e** Finally, a machine learning model can be used to combine the biomarkers for a regression or classification task.

Because respiratory conditions may possess different oximetry patterns/dynamics and oximetry recordings may be of different durations, it is important to define a general methodology for continuous oximetry time series analysis using the OBM toolbox. The suggested flow for such analysis is illustrated in Fig. 3. Following these steps, performance statistics relevant to the task at hand should be reported and a clear discussion should be delivered regarding the biomarkers that were most relevant to the data-driven model including interpretation about the underlying physiology.

Oximetry biomarkers may vary significantly with the technology used (transmission versus reflectance) as well as by the manufacturer. Most oximeters use two light-emitting diodes (LED) that face a translucent part of the body, such as the fingertip or earlobe, and a photodiode that receives light rays. In most cases, one LED is red and the second infrared. The oximeter includes a processor that calculates the oxygen saturation using the ratio between the amount of light that was emitted and the amount that was received at each wavelength. Oximeters may be transmissive or reflective. In a transmissive oximeter, the photodiode and the LEDs are placed on opposite sides of the measurement site and the light passes through the site. In a reflective oximeter, the LEDs are placed on the same side and the light is reflected to the photodiode across the measurement site.

During the current coronavirus disease 2019 (COVID-19) pandemic, many individuals with suspected or confirmed, but mild, COVID-19 are told to monitor their symptoms at home or from government-managed locations. Hospitalization is only an option if there is a medical need. Monitoring the blood oxygen level may be a meaningful way to remotely monitor individuals with mild COVID-19^21^. It could also be used for continuous monitoring of patients in the ICU with pneumonia, a common complication of COVID-19. However, there is a lack of smart algorithms that can exploit the information encrypted within these oxygen saturation physiological time series. The development of such algorithms will facilitate the continuous monitoring of COVID-19 ICU patients in predicting deteriorations. It remains to be determined how the information contained in the oxygen saturation physiological time series can be exploited. Are trends or absolute values or the occurrence of specific patterns the most meaningful information for identification of the disease and prediction of its course? The pobm toolbox developed in this publication can support researching novel biomarkers for diagnosis and prognosis of COVID-19.

Additional OBMs, such as kernel entropy, bispectrum, and wavelet, among others, should be considered and added to the library in future works. Although we demonstrated the usage of the PhysioZoo OBM resource on OSA, there is a need to assess the value of these biomarkers for other respiratory disorders.

Typical oximetry biomarkers used in clinical practice include the ODI and CT90. While these indices are standardized, to some extent, and interpretable, they fail to capture important pathophysiological characteristics. We reviewed evidence-based oximetry biomarkers, suggested a classification system, and created a unique resource (pobm toolbox and PhysioZoo OBM interface) for performing oximetry time series analysis. This resource can be applied to gain novel physiological, clinical, and epidemiological insights.

## Methods

### Oximetry biomarker categorization

Various categorizations of OBMs have been previously suggested^10,22^. We introduce a five-category classification scheme of our own, which we believe best reflects the literature and usage of these biomarkers in medical practice.

(i) *General statistics:* these are time-based statistics describing the oxygen saturation time series data distribution.
(ii) *Complexity*: quantifies the presence of long-range correlations in non-stationary time series.
(iii) *Periodicity*: quantifies consecutive events to identify periodicity in the oxygen saturation time series.
(iv) *Desaturations:* time-based descriptive measures of the desaturation patterns occurring throughout the time series.
(v) *Hypoxic burden*: time-based measures quantifying the overall degree of hypoxemia imposed on the heart and other organs during the recording period.

A comprehensive summary of the OBMs reviewed and implemented in this research is presented in Table 3 for the general statistics, complexity, and periodicity categories and in Table 4 for the desaturation measures and hypoxic burden categories. A total of 44 oximetry biomarkers were engineered. A glossary with variables symbols and definition is presented in Supplementary Table 3.

**Table 3:** List of digital oximetry biomarkers for the categories: general statistics, complexity, and periodicity.

	Biomarker	Definition	Unit
*General statistics*			
1	AV	Blood oxygen saturation (SpO_2_) mean	%
2	MED	SpO_2_ median	%
3	Min	SpO_2_ min	%
4	SD	SpO_2_ standard deviation	%
5	RG	SpO_2_ range	%
6	Px	*x*^th^ percentile SpO_2_ value, by default *x* = 1	%
7	Mx	Percentage of the signal at least *x*% below median oxygen saturation, by default *x* = 2, used by Deviaene et al.^22^	%
8	ZCx	Number of zero-crossing points at the *x*% SpO_2_ level^25^, by default *x* = AV	nu
9	ΔIx	Delta index^26^, by default *x* = 12 s.	%
*Complexity*			
10	ApEn	Approximate entropy^28^ with, by default, *m* = 1, *r* = 0.25 times the standard deviation of the data	nu
11	LZ	Lempel–Ziv complexity^31^	nu
12	CTM_*ρ*_	Central tendency measure^37^ with radius *ρ*, by default *ρ* = 0.25	nu
13	SampEn	Sample entropy^29^ with, by default, *m* = 1, *r* = 0.25	nu
14	DFA	Detrended fluctuation analysis^36^ with, by default, *n* = 20	%
*Periodicity*			
15	PRSAD_c_	Phase-rectified signal averaging (PRSA) capacity^22,38^. With *d* the fragment duration, by default *d* = 10	%
16	PRSAD_ad_	PRSA amplitude difference^22,38,57^. With *d* the fragment duration, by default *d* = 10	%
17	PRSAD_os_	PRSA overall slope^38,57^. With *d* the fragment duration, by default *d* = 10	%/s
18	PRSAD_sb_	PRSA slope before the anchor point^38,57^. With *d* the fragment duration, by default *d* = 10	%/s
19	PRSAD_sa_	PRSA slope after the anchor point^38,57^. With *d* the fragment duration, by default *d* = 10	%/s
20	AC	Autocorrelation	%^2^
21	PSD_total	The integral of the power spectral density (PSD) function^45^	%
22	PSD_band	The integral of the PSD function within the band 0.014−0.033 Hz^45^	%
23	PSD_ratio	The integral of the PSD function within the band 0.014−0.033 Hz with respect to the total integral^45^	nu
24	PSD_peak	Peak amplitude of the PSD function within the band 0.014−0.033 Hz^45^	%

**Table 4:** List of digital oximetry biomarkers for the categories: desaturation measures and hypoxic burden.

	Biomarker	Definition	Unit
*Desaturation measures*			
25	ODI_*x*_	The oxygen desaturation index as defined in Jung et al. and Behar et al.^50,51^, by default *x* = 3	event/h
26	DL_*μ*_	Mean of desaturations length (e.g., related work in Kulkas et al.^47^)	s
27	DL_*σ*_	Standard deviation of desaturations length (e.g., related work in Kulkas et al.^47^)	s^2^
28	DDmax_*μ*_	Mean of desaturations depth (e.g., related work in Kulkas et al.^47^)	%
29	DDmax_*σ*_	Standard deviation of desaturation depth (e.g. related work in Kulkas et al.^47^)	%^2^
30	DD100_*μ*_	Mean of desaturations depth using 100% SpO_2_ level as baseline (e.g., related work in Terrill et al.^10^)	%
31	DD100_*σ*_	Standard deviation of desaturations depth using 100% SpO_2_ level as baseline (e.g., related work in Terrill et al.^10^)	%^2^
32	DS_*μ*_	Mean of the desaturation slope	%/s
33	DS_*σ*_	Standard deviation of the desaturation slope	(%/s)^2^
34	DAmax_*μ*_	Desaturation area defined as the mean of the desaturation areas using the maximum SpO_2_ value in each desaturation event as baseline^47^	%*s
35	DAmax_*σ*_	Standard deviation of desaturation area^47^	(%*s)^2^
36	DA100_*μ*_	Desaturation area: mean of desaturation area under the 100% SpO_2_ level as baseline^47^	%*s
37	DA100_*σ*_	Standard deviation of desaturation area under the 100% SpO_2_ level as baseline^47^	(%*s)^2^
38	TD_*μ*_	Mean of time between two consecutive desaturation events	sec
39	TD_*σ*_	Standard deviation of time between two consecutive desaturation events	s^2^
*Hypoxic burden*			
40	POD_*x*_	Time of oxygen desaturation event, normalized by the total recording time^18^, by default *x* = 3	s
41	AOD_max_	The area under the oxygen desaturation event curve^18^, using the maximum SpO_2_ value as baseline and normalized by the total recording time	%
42	AOD_100_	Cumulative area of desaturations under the 100% SpO_2_ level as baseline and normalized by the total recording time	%
43	CT_*x*_	Cumulative time below the *x*% oxygen saturation level, by default *x* = 90. Introduced by Olson et al.^55^	%
44	CA_*x*_	Integral of SpO_2_ below the *x* SpO_2_ level normalized by the total recording time, by default *x* = AV	%

### Preprocessing

Raw oximetry data is often associated with missing values and artefacts caused, for example, by motion of the oximeter or lack of proper contact between the finger and the probe. Therefore, the toolbox includes an option for two preprocessing filters:

#### Delta filter

**Fig. 4:**
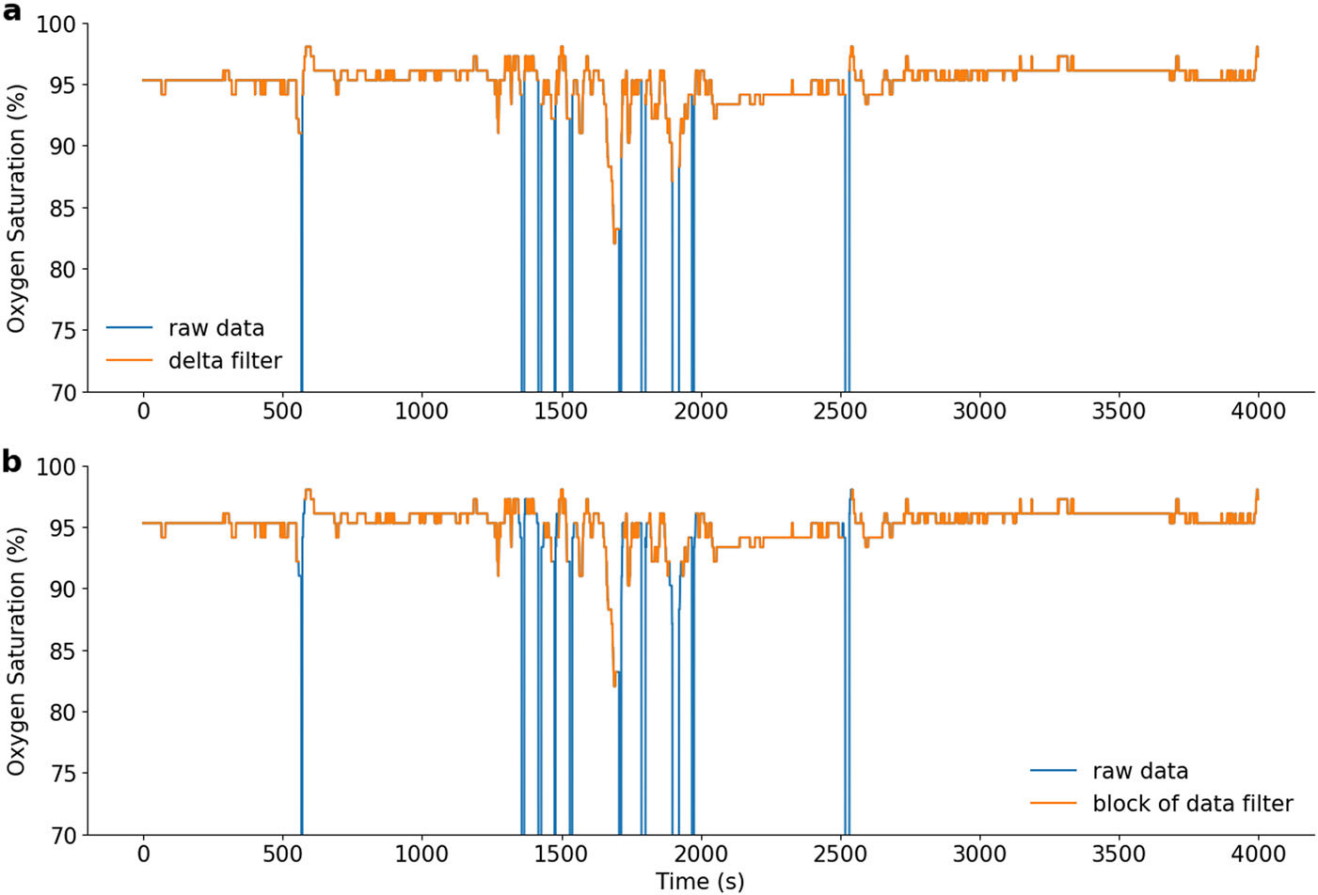
Example of preprocessing approaches. **a** shows the time series preprocessed with the delta filter technique, and **b** shows the same time series preprocessed with the block of data technique.

A delta filter is applied to the SpO_2_ time series, in which, when two consecutive samples are >*x*%/s apart, they are considered non-physiological and are discarded. By default, *x* = 4%/s apart as in the work of Taha et al.^23^. As an example, applying the delta filter to the SpO_2_ time series is shown in Fig. 4a.

#### Block of data filter

An error value is considered a value <50%. For each error value, a small block of data of length *x* s (default is *x* = 20 s) around it is discarded. Once the small blocks are removed, the mean is computed for each block of data of length 100 s around the original error value. The mean of the overall SpO_2_ signal is also computed. Each block with a mean <6% smaller than the overall mean is discarded. This technique was used by Buekers et al.^24^. As an example, applying the block of data filter to the SpO_2_ time series is shown in Fig. 4b.

### General statistics

Average (AV): average of SpO_2_ values. Median (MED): median of SpO_2_ values. Min (Min): minimum of SpO_2_ values representing the physiological minimum of the SpO_2_. Standard deviation (SD): standard deviation of SpO_2_ values. Range (RG): the difference between the maximal and minimal SpO_2_ values. Percentile (Px): The *x*th percentile of SpO_2_ values. BelowMedian (Mx): Percentage of the signal *x*% below median oxygen saturation.

#### ZeroCrossing (ZCx)

ZeroCrossing (ZCx) is the number of zero-crossing points, used by Xie et al.^25^, using the *x*% SpO_2_ level as baseline. A crossing point is considered as two consecutive samples of the SpO_2_ signal, one lower than the baseline and the second greater, or vice versa. This biomarker helps to understand how the signal oscillates around a baseline. The intuition is that a SpO_2_ time series from a patient with OSA as compared to that of a non-OSA patient will oscillate more around the baseline because of the presence of desaturations and then reach a higher value. A common baseline used for this biomarker is the mean of the signal (i.e., by default, *x* = AV). ZCx is defined as:

1 $${\mathrm{ZCx}} = \mathop {\sum}\limits_{i = 10}^{N_{{\mathrm{SpO}}_2} - 1} {{\mathrm{ZC}}_i\left( x \right)},$$

2 $${\mathrm{ZC}}_i\left( x \right) = \left\{ {\begin{array}{ll} 1 & {\mathrm{if}}\left( {{\mathrm{SpO}}2_i - x} \right)\left( {{\mathrm{SpO}}2_{i + 1} - x} \right) < \, 0 \\ 0 & {\mathrm{else}} \end{array}}, \right.$$

where *N*_SpO2_ is the number of samples of the SpO_2_ time series.

#### Delta index (ΔIx)

ΔIx^26^ corresponds to the sum of the absolute variations between two successive points divided by the number of intervals. The original intuition was that SpO_2_ oscillations, induced by repeated apnea resumption of ventilation sequence, will lead to a high ΔI, while COPD-induced prolonged desaturations or nearly constant SpO_2_ would lead to a low ΔI. In the original paper of Pepin et al.^26^, recordings from a total of 160 consecutive patients referred for PSG were used to set the threshold of the ΔI distinguishing between OSA and non-OSA. When testing on the prospective group of patients, i.e., *n* = 36 patients with *p* = 34 nights of recordings for each patient, they obtained a sensitivity of 0.75 and specificity of 0.86. In Magalang et al.^27^, the ΔI was the best predictor (*r*^2^ = 0.60) for the AHI versus other OBMs including the ODI. The ΔI index is defined as:

3 $$\Delta {\mathrm{Ix}} = \frac{1}{{N_{{\mathrm{window}}}}} \cdot \mathop {\sum}\limits_{i = 1}^{N_{{\mathrm{window}}}} {\left| {{\mathrm{SpO}}2\_{\mathrm{window}}_{i + 1} - {\mathrm{SpO}}2\_{\mathrm{window}}_i} \right|},$$

where SpO2_window_*i*_ is the average of the level of oxygen saturation for the window *i* of length *x* s, and *N*_window_ is the number of windows. In their original work, Pepin et al.^26^ used *x* = 12 s. In our implementation, signals are re-sampled to 1 Hz by default, so, by default, a window will contain 12 samples.

### Complexity measures

Regularity quantifies how often similar patterns are observed in the oximetry signal^10^. In the context of physiological time series analysis, approximate entropy (ApEn)^28^ and sample entropy (SampEn)^29^ have commonly been used as measures of the unpredictability (opposite of regularity). OSA individuals typically have less regular oximetry patterns, leading to higher ApEn and SampEn values as compared to non-OSA individuals. Loss of physiological complexity may be better captured by using other measures that can detect and quantify the presence of long-range correlations in non-stationary time series^30^ with measures such as the Lempel–Ziv complexity (LZ)^31^. Fractal objects, generated by stochastic or nonlinear deterministic mechanisms^30^, may also be used to capture complexity, as they show self-similarity, i.e., the smaller-scale structure resembles the larger-scale form^32^. Detrended fluctuation analysis (DFA) has commonly been used for fractal analysis in the field of physiological time series analysis.

#### Approximate entropy

ApEn is a biomarker introduced in Pincus et al.^28^, which aims to capture the irregularity in the signal, with higher values indicating higher irregularity. This biomarker is very useful in the detection of OSA, as high randomness is associated with high values of the biomarker. Thus apneas and hypopneas are associated with high ApEn values. ApEn(*m*, *r*, *N*) can be defined as:

4 $${\mathrm{ApEn}} = {\upvarphi}^m\left( r \right) - {\upvarphi}^{m + 1}\left( r \right),$$

5 $${\upvarphi}^m\left( r \right) = \frac{1}{{N - m + 1}}\mathop {\sum}\limits_{i = 1}^{N - m + 1} {\ln \left( {\frac{{N^m\left( i \right)}}{{\left( {N - m + 1} \right)}}} \right)},$$

where *N*^*m*^(*i*) is the number of windows of length *m* for which the distance from the window beginning at the index *i* is lower than or equal to *r*. The distance between two windows can be defined as:

6 $$d\left( {X\left( i \right),\,X\left( j \right)} \right) = \mathop {{\max }}\limits_{1 \le k \le m} \left| {x\left( {i + k - 1} \right) - x\left( {j + k - 1} \right)} \right|.$$

This biomarker was first used in the context of OSA diagnosis from oximetry data, in a study by Hornero et al.^33^, with *m* = 1, *r* = 0.25·*σ*, where *σ* is the standard deviation of the data. The database was composed of SpO_2_ time series from subjects showing symptoms of sleep disordered breathing categorized into OSA-positive and OSA-negative groups according to the gold standard PSG. ApEn was used to diagnose OSA and reached 82.09% sensitivity and 86.96% specificity on a test set composed of *n* = 113 individuals.

#### Sample entropy

This is a non-linear biomarker that quantifies the irregularity in the data and has less bias compared to ApEn. It was used in the original work of Richman and Moorman^29^, who proved the robustness of sample entropy within the context of physiological time-series analysis (on neonatal HRV). This biomarker has also been used by Behar et al.^6^ for HRV analysis across different mammals. A pseudo-code for the implementation of SampEn is provided in Supplementary Methods.

#### LZ complexity

This biomarker was introduced by Lempel and Ziv^31^ in 1976. Within the context of SpO_2_ analysis, LZ evaluates the degree of complexity of spatiotemporal patterns in the SpO_2_ signal. It has been largely used in the domain of medicine, especially in the domain of biomedical signal analysis, such as in the work of Amigó et al.^34^, who used it on electroencephalogram time series, or by Álvarez et al.^35^ to discriminate between OSA and non-OSA individuals. For the later work, it resulted in a sensitivity of 86.5%, a specificity of 77.6%, and an accuracy of 82.9%, when tested on a population of *n* = 187 patients, including 147 males and 40 females. A pseudo-code for the LZ measure is available in Supplementary Methods.

#### Detrended fluctuation analysis

DFA is a scaling analysis method that aims to represent the autocorrelation properties of the signal. A major advantage of this method is its robustness against non-stationarity of the signal. This biomarker was introduced by Peng et al.^36^ to identify crossover behavior in signals. Larger fluctuations typical of repetitive desaturations lead to a higher DFA profile, while near-constant or slow, longer desaturations result in lower profiles. A pseudo-code for the implementation of DFA is provided in Supplementary Methods.

#### Central tendency measure (CTM_*ρ*_)

CTM_*ρ*_ is a non-linear method first proposed by Cohen et al.^37^, with the goal of assessing the degree of variability in cardiac physiological data. The higher the variability in the SpO_2_ signal (i.e., more desaturations/apneas), the lower the CTM_*ρ*_. Indeed, as CTM_*ρ*_ measures the number of points within a circular region of radius *ρ*, the higher the variability/dispersion the lower the number of points within the circle so the lower the CTM_*ρ*_. This biomarker was used in the study of Álvarez et al.^35^ on a dataset composed of *n* = 187 patients, with *ρ* = 0.25 for the purpose of OSA diagnosis. Their analysis resulted in a sensitivity of 90.01% and a specificity of 82.9%. CTM_*ρ*_ is calculated as:

7 $${\mathrm{CTM}}_\rho = \frac{{\mathop {\sum}\nolimits_{i = 1}^{N_{{\rm{SpO}}_2} - 2} {\delta \rho \left( i \right)} }}{{N_{{\rm{SpO}}_2} - 2}},$$

8 $$\begin{array}{l}\delta \rho \left( i \right) =\\\quad \left\{ \begin{array}{ll} 1 & {\mathrm{if}}\,\sqrt {\left( {{\mathrm{SpO}}_2\left( {i + 2} \right) - {\mathrm{SpO}}_2\left( {i + 1} \right)} \right)^2 + \left( {{\mathrm{SpO}}_2\left( {i + 1} \right) - {\mathrm{SpO}}_2\left( i \right)} \right)^2}\, < \,\rho \\ 0 & {\mathrm{else}} \end{array} \right.\end{array}.$$

### Periodicity measures

Consecutive apneic events create some periodicity in the oxygen saturation time series. This periodicity can be quantified through techniques, such as frequency analysis, phase-rectified signal averaging (PRSA), and autocorrelation.

#### Phase-rectified signal averaging

**Fig. 5:**
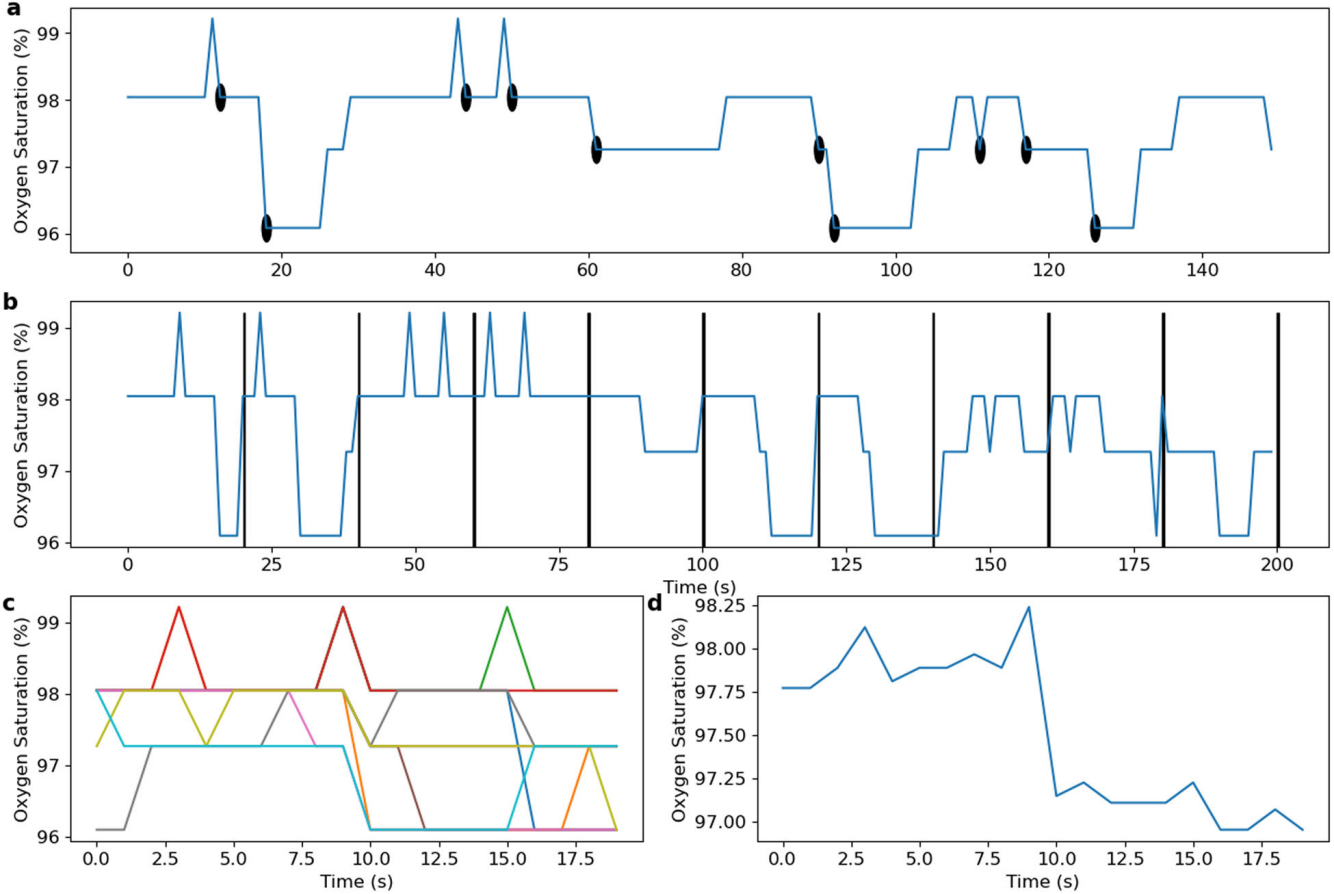
PRSA analysis of the oximetry time series. In **a**, the oximetry time series, with anchor points highlighted. In **b**, concatenation of PRSA windows, defined around each anchor points. In **c**, PRSA windows of top of each other. In **d**, the average of all the PRSA windows.

PRSA is a signal processing technique introduced by Bauer et al.^38^ to detect and quantify quasi-periodic oscillations in a noisy non-stationary signal. The method also identifies patterns in increasing and decreasing regions of the signal. A PRSA window can be defined as:

9 $$\begin{array}{*{20}{c}} {\overline x \left( k \right) = \frac{1}{M}\mathop {\sum}\limits_{i = 1}^M {X_i\left[ k \right],} } & {{\mathrm{for}} - {L}\, \le \,{k}\,<\,{L}} \end{array},$$

where *X*_*i*_ is the window of length 2*L* around the anchor point *x*(*i*) and *M* is the number of anchor points. An anchor point is a decreasing point in the signal: *x*(*i*) < *x*(*i*−1), such that the decreasing part (negative slope) of the desaturation is always within the window. It can also be defined as increasing points in order to investigate patterns in the resaturation part of the event. Figure 5 shows an example of PRSA computation on an oximetry time series for *L* = 10 and *M* = 10 anchor points.

Within the context of OSA diagnosis, PRSA biomarkers were used in the study of Deviaene et al.^22^ and evaluated on three datasets: the Sleep Heart Health Study (SHHS) dataset^39–42^, the Apnea-ECG^43^ dataset, and a third set recorded at the sleep laboratory of the University Hospital Leuven. Five PRSA biomarkers were found significant and these are the ones that we implemented ^22^.

- PRSAd_c_, the capacity of the window, defined as
  10 $${\mathrm{PRSAd}}_{\mathrm{c}} = \frac{{\overline x \left( 0 \right) + \overline x \left( 1 \right) - \overline x \left( { - 1} \right) - \overline x \left( { - 2} \right)}}{4}.$$
- PRSAd_ad_, the amplitude differences; this is the difference between max and min values.
- PRSAd_os_, the overall slope of the window; the window is linearly approximated, and the slope is retained.
- PRSAd_sb_, the slope before the anchor point.
- PRSAd_sa_, the slope after the anchor point.

#### Autocorrelation

AC(*k*) measures the degree of correlation between values of the same variable. This is achieved by computing the correlation between the original SpO_2_ time series and a shifted version of it. The analysis of AC can be used to find repeating patterns, such as a periodic signal. Mathematically, AC can be defined as:

11 $${\mathrm{AC}}\left( k \right) = \mathop {\sum}\limits_{i = 1}^{N - k} {{\mathrm{SpO}}_2\left( i \right) \ast {\mathrm{SpO}}_2\left( {i + k} \right)}.$$

#### Power spectral analysis (power spectral density (PSD_total_, PSD_band_, PSD_ratio_, PSD_peak_)

**Fig. 6:**
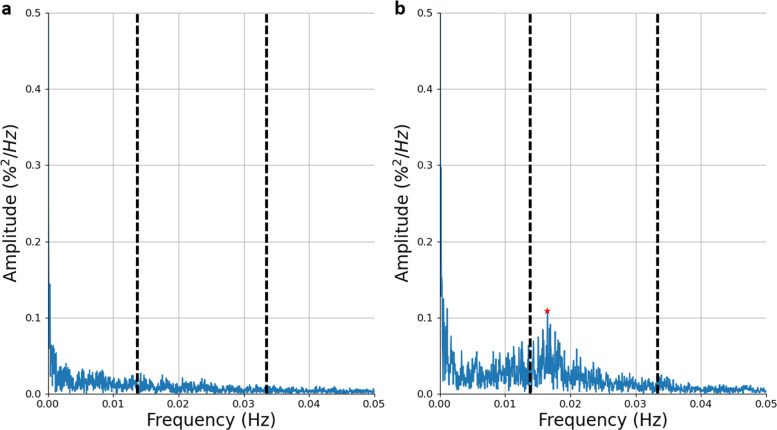
Power spectral density (PSD) of the oximetry time series. Example on **a** a non-OSA individual and **b** a patient with moderate OSA. The band 0.014–0.033 Hz is delineated by the vertical dashed lines. In **b**, the peak within this band is located at *f* = 0.017 Hz, as highlighted by the red star.

In Zamarrón et al.^44^, the authors analyzed the PSD curve of the oximetry time series. They defined a spectral band of interest for oximetry analysis within the context of OSA as 0.014–0.033 Hz. Zamarrón et al.^45^ assessed PSD biomarkers on a total of 250 subjects between the ages of 21 and 82 years and obtained a sensitivity of 78.2% and a specificity of 89.0%. Figure 6 shows the differences in the spectral signal between a non-OSA and an OSA patient.

- PSD_total_ corresponds to the area defined by the power spectrum:

12 $${\mathrm{PSD}}_{{\mathrm{total}}} = \mathop {\sum}\limits_{i = 1}^{{\mathrm{NFFT}}} {X\left( i \right)},$$

where *X* is the amplitude of the PSD function, estimated by the Welch’s method^46^ using a hamming window, and NFFT is the number of points in the PSD signal.

- PSD_band_ corresponds to the energy within the band 0.014–0.033 Hz:

13 $${\mathrm{PSD}}_{{\mathrm{band}}} = \mathop {\sum}\limits_{i = N_1}^{N_2} {X\left( i \right),}$$

where *N*_1_ and *N*_2_ are the limits of the summation between 0.014 and 0.033 Hz.

- PSD_ratio_ corresponds to the ratio between the power (area) within the spectral band 0.017–0.033 Hz and PSD_total_.
  14 $${\mathrm{PSD}}_{{\mathrm{ratio}}} = \frac{{\mathop {\sum}\nolimits_{i = N_1}^{N_2} {X\left( i \right)} }}{{\mathop {\sum }\nolimits_{i = 0}^{{\mathrm{NFFT}}} X\left( i \right)}},$$
- PSD_peak_ corresponds to the peak amplitude of the PSD within the band 0.014–0.033 Hz.

15 $${\mathrm{PSD}}_{{\mathrm{peak}}} = \mathop {{{\mathrm{max}}}}\limits_{N_1 \,< \,i\, <\, N_2} \left\{ {X\left( i \right)} \right\},$$

where *i* is the index of the power spectrum signal.

### Desaturation measures

Desaturations can occur as a consequence of conditions such as sleep disordered breathing, and can be characterized by descriptors such as their lengths and depths. For example, a study by Kulkas et al.^47^ studied the gender difference in the distribution of the desaturation lengths, depths, and areas caused by hypopnea and apnea events. Desaturations are not only caused by apnea or hypopnea events^48^ and thus desaturation events and their statistical descriptors may capture the expression of other conditions during sleep.

#### Oxygen desaturation index (ODI_*x*_)

**Fig. 7:**
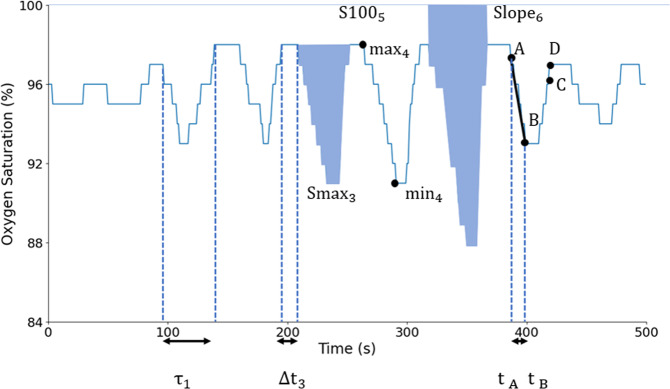
Desaturation biomarkers. A, B, C, and D are the fiducials points delimiting a desaturation event.

The ODI_*x*_ corresponds to the average number of desaturation events per hour. A desaturation is defined as a SpO_2_ drop of *x*% below the baseline. The ODI_*x*_ is a widely used measure in the field of sleep medicine, where desaturations are characteristic of apnea and hypopnea events^49^. Indeed, obstruction of the airway leads to reduced entrance of oxygenated air to the lungs, which leads to a drop in oxygenated hemoglobin until airway patency is restored. These manifest as transient hypoxemic events or desaturations. Traditionally *x* = 3 or 4%. There exist many implementations of the ODI with variable definitions. In the present work, the implementation of the ODI_*x*_ detection algorithm was developed and validated by Behar et al.^50^, building on the parent model and desaturation definition of Jung et al.^51^. Specifically, the model of Jung et al.^51^ defines three fiducial points A, B, and C, to determine the occurrence of a desaturation. Fiducial point A is defined as the point where the SpO_2_ value decreases by ≥1 and ≤3%, fiducial point B as the value that reaches a minimum of at least 3% below A, and fiducial point C as the point where the SpO_2_ value returns to a level either 1% below A or 3% above B. Some additional constraints are imposed, including that fluctuations in consecutive SpO_2_ values should be <1% between A and B and >−1% between B and C. Finally, the time interval between A and C must be ≥10 and ≤60 s^51^. In the original paper, 90 s was used as the time limit, but to ensure capture of resaturation, 60 s is used here. An additional fiducial point D was defined as a point posterior to C at which the SpO_2_ time series reaches a level of at least 1% below A and where the time interval between A and D is ≤60 s. From the detected desaturation events, several oximetry biomarkers can be computed (Fig. 7). Within our context of single-channel SpO_2_ analysis, i.e., when no reference EEG channel is available to quantify sleep time, the ODI_*x*_ is defined as:

16 $${\mathrm{ODI}}_x = \frac{{N_{{\mathrm{desat}}}}}{{{\mathrm{TRT}}}},$$

where *N*_desat_ is the number of desaturations in the signal and TRT is the total recording time in hours.

#### Desaturation length (DL_*μ*_, DL_*σ*_)

DL_*μ*_ is the mean and DL_*σ*_ is the standard deviation across the entire length of the desaturation. These biomarkers contain information about the duration of the desaturation events in the SpO_2_ signal. Indeed, ODI_*x*_ only considers the number of desaturations but does not consider their length. The length of a desaturation is particularly important because it reflects how long an individual is under hypoxic stress. Furthermore, desaturations of various lengths will lead to a higher DL_*σ*_. DL_*μ*_ and DL_*σ*_ are defined as:

17 $${\mathrm{DL}}_{\mu} = \frac{1}{{N_{{\mathrm{desat}}}}}\mathop {\sum}\limits_{i = 1}^{N_{{\rm{desat}}}} {{\tau }}_i,$$

18 $${\mathrm{DL}}_{\sigma} = \sqrt {\frac{{\mathop {\sum}\nolimits_{i = 1}^{N_{{\mathrm{desat}}}} {\left( {{\tau }}_i - {\mathrm{DL}}_{\mu} \right)^2} }}{{N_{{\mathrm{desat}}}}}},$$

where *τ*_*i*_ corresponds to the duration of the *i*th oxygen desaturation event.

#### Desaturation depth (DDmax_*μ*_, DDmax_*σ*_, DD100_*μ*_, DD100_*σ*_)

For a single desaturation, the desaturation depth is computed as the maximal minus the minimal SpO_2_ value within the desaturation event, from point A to B. DDmax_*μ*_ is the mean and DDmax_*σ*_ is the standard deviation across all individual desaturation depths. A similar pair of biomarkers can be engineered by computing the depth with respect to the 100% SpO_2_ level, i.e., 100% minus the minimal SpO_2_ value of a desaturation event. These are, respectively, denoted DD100_*μ*_ and DD100_*σ*_ for the mean and the standard deviation computed across all desaturation depths. The idea of quantifying the desaturation depth has also been used by Terrill^10^. The desaturation depth may be an important factor for determination of the severity of OSA because it will reflect, for a given desaturation, the level of the hypoxic stress imposed. Desaturation depth also varies with sleep stages, and so there is value in capturing its variation across desaturations^52^. Furthermore, alternation of soft and deep desaturations will lead to high values of DDmax_*σ*_ and DD100_*σ*_. The two pairs of biomarkers are defined as:

19 $${\mathrm{DDmax}}_{\mu} = \frac{1}{{N_{{\mathrm{desat}}}}}\mathop {\sum}\limits_{i = 1}^{N_{{\mathrm{desat}}}} {{\mathrm{max}}_i - {\mathrm{min}}_i},$$

20 $${\mathrm{DD}}100_{\mu} = \frac{1}{{N_{{\mathrm{desat}}}}}\mathop {\sum}\limits_{i = 1}^{N_{{\mathrm{desat}}}} {100 - {\mathrm{min}}_i},$$

21 $${\mathrm{DDmax}}_{\sigma} = \sqrt {\frac{{\mathop {\sum }\nolimits_{i = 1}^{N_{{\mathrm{desat}}}} \left( {{\mathrm{max}}_i - {\mathrm{min}}_i - {\mathrm{DDmax}}_{\mu}} \right)^2}}{{N_{{\mathrm{desat}}}}}},$$

22 $${\mathrm{DD}}100_{\sigma} = \sqrt {\frac{{\mathop {\sum}\nolimits_{i = 1}^{N_{{\mathrm{desat}}}} {\left( {100 - {\mathrm{min}}_i - {\mathrm{DD}}100_{\mu}} \right)^2} }}{{N_{{\mathrm{desat}}}}}},$$

where max_*i*_ is the maximum value of desaturation *i* and min_*i*_ is the minimum value of desaturation *i*. The variables max_*i*_ and min_*i*_ are illustrated in Fig. 7. The desaturation depth can also be described by extending the ODI to any threshold *x* and studying the cumulative frequency of the desaturations as a function of *x*^53^.

#### Desaturation slope (DS_*μ*_, DS_*σ*_)

The downslope of the signal is calculated for each desaturation. The decreasing phase of the desaturation is linearly approximated. DS_*μ*_ is the mean and DS_*σ*_ is the standard deviation of the slopes over all desaturation events. These biomarkers consider the slope of the desaturation, which is a different factor than the number, duration, or depth of the desaturations. Indeed, OSA and other pathologies may lead to sharp drops in SpO_2_, which would lead to high DS_*μ*_ value. The slope of a specific desaturation can be written as:

23 $${\mathrm{Slope}}_i = \frac{{B - A}}{{t_B - t_A}},$$

where (*A*, *t*_*A*_) is the point of inflexion (amplitude and timestamp) of the desaturation, and (*B*, *t*_*B*_) is the minimum point of the desaturation. Accordingly, the mean and standard deviation of slopes are computed as:

24 $${\mathrm{DS}}_{\mu} = \frac{1}{{N_{{\mathrm{desat}}}}}\mathop {\sum}\limits_{i = 1}^{N_{{\mathrm{desat}}}} {{\mathrm{Slope}}_i},$$

25 $${\mathrm{DS}}_{\sigma} = \sqrt {\frac{{\mathop {\sum}\nolimits_{i = 1}^{N_{{\mathrm{desat}}}} {\left( {{\mathrm{Slope}}_i - {\mathrm{DS}}_{\mu}} \right)^2} }}{{N_{{\mathrm{desat}}}}}}.$$

Slope_*i*_ can be seen in Fig. 7.

#### Desaturation area (DAmax_*μ*_, DAmax_*σ*_, DA100_*μ*_, DA100_*μ*_)

The area of the desaturation is computed for each desaturation. DA100_*μ*_ is the mean and DA100_*σ*_ is the standard deviation of the area across all individual desaturations, taking 100% SpO_2_ as baseline. The area can also be computed by taking the maximum SpO_2_ value of individual desaturations as baseline. DAmax_*μ*_ is the mean and DAmax_*σ*_ is the standard deviation of the area across all individual desaturations. Whereas the *Desaturation length* biomarker considers the duration of the events (reflecting the time under hypoxic stress) and the *desaturation depth* biomarkers consider the depth of events (reflecting the strength of hypoxia), the desaturation area factorizes both the depth and the length of the desaturations. The two pairs of biomarkers can be mathematically written as:

26 $${\mathrm{DA}}100_{\mu} = \frac{1}{{N_{{\mathrm{desat}}}}}\mathop {\sum}\limits_{i = 1}^{N_{{\mathrm{desat}}}} {{\mathrm{S}}100_i},$$

27 $${\mathrm{DA}}100_{\sigma} = \sqrt {\frac{{\mathop {\sum}\nolimits_{i = 1}^{N_{{\mathrm{desat}}}} {\left( {{\mathrm{S}}100_i - {\mathrm{DA}}100_{\mu}} \right)^2} }}{{N_{{\mathrm{desat}}}}}},$$

28 $${\mathrm{DAmax}}_{\mu} = \frac{1}{{N_{{\mathrm{desat}}}}}\mathop {\sum}\limits_{i = 1}^{N_{{\mathrm{desat}}}} {{\mathrm{Smax}}_i},$$

29 $${\mathrm{DAmax}}_{\sigma} = \sqrt {\frac{{\mathop {\sum}\nolimits_{i = 1}^{N_{{\mathrm{desat}}}} {\left( {{\mathrm{Smax}}_i - {\mathrm{DAmax}}_{\mu}} \right)^2} }}{{N_{{\mathrm{desat}}}}}},$$

where Smax_*i*_ is the area of the specific desaturation event integrated from the maximal (max) value of the desaturation event and S100_*i*_ is the area of the specific desaturation event integrated from 100%. Smax_*i*_ and S100_*i*_ can be seen in Fig. 7.

#### Time between desaturation (TD_*μ*_, TD_*σ*_)

The average and standard deviation of time elapsed between two consecutive desaturation events can be used to capture some aspect of the temporal distribution of desaturation events. The two biomarkers can be computed as:

30 $${\mathrm{TD}}_{\mu} = \frac{1}{{N_{{\mathrm{desat}}} - 1}}\mathop {\sum}\limits_{{\mathrm{i}} = 2}^{N_{{\mathrm{desat}}}} {\Delta t_i},$$

31 $${\mathrm{TD}}_{\sigma} = \sqrt {\frac{{\mathop {\sum}\nolimits_{i = 2}^{N_{{\mathrm{desat}}}} {\left( {\Delta t_i - {\mathrm{TD}}_{\mu}} \right)^2} }}{{N_{{\mathrm{desat}}} - 1}}},$$

where Δ*t*_*i*_ is the time elapsed between desaturation *i* and desaturation *i* − 1. Δ*t*_*i*_ can be seen in Fig. 7.

### Measures of the hypoxic burden

#### The percentage of oxygen desaturation events (POD_*x*_)

The POD_*x*_ is the overall duration of all desaturations, normalized by the total recording time. It was introduced by Kulkas et al.^18^ in order to estimate the severity of OSA from the SpO_2_ time series. It was used in the work of Watanabe et al.^54^ to study the prognostic importance of novel oxygen desaturation measures in heart failure and central sleep apnea population samples. Non-survivors had a higher POD_*x*_ compared with survivors (19 ± 13 versus 11 ± 6.4%; *p* = 0.001). By contrast, non-survivors did not differ significantly from survivors with respect to the AHI and CT90%. An adjusted logistic regression analysis revealed that the POD_*x*_ was the best independent predictor of mortality. In the work by Kulkas et al.^18^, the biomarker was computed on a dataset collected from 160 male patients with different levels of AHI severity. The correlation between AHI and POD_*x*_ was high: *r*^2^ = 0.87^18^. The PODx can be mathematically defined as:

32 $${\mathrm{POD}}_x = 100 \cdot \frac{{\mathop {\sum}\nolimits_{i = 1}^{N_{{\mathrm{desat}}}} {{\tau }}_i }}{{{\mathrm{TRT}}}},$$

where *τ*_i_ (Fig. 7) corresponds to the duration of each oxygen desaturation event and *x* to the level of the desaturation. In their original publication, Watanabe et al.^54^ set *x* = 4%.

#### The area under the oxygen desaturation curve (AODmax, AOD100)

The AOD_*x*_ was introduced by Kulkas et al.^18^ in the same context as the POD_*x*_, for the estimation of the sleep apnea–hypopnea syndrome. It was used in the work of Watanabe et al.^54^, along with the POD biomarker. Survivors in this study appeared to have lower AOD_*x*_ than non-survivors (0.16 ± 0.2 versus 0.26 ± 0.2; *p* = 0.08). It represents the sum of the area of each desaturation event divided by TRT. This index was demonstrated to be an independent modulator of increased epicardial fat volume (EFV) in an acute myocardial infarction population sample^14^ (*n* = 105). EFV is associated with adverse cardiovascular events after myocardial infarction. In the work of Kulkas et al.^18^, this biomarker appeared to have moderate correlation with AHI: *r*^2^ ∈ [0.581−0.689], *p* < 0.001. It can be mathematically defined as:

33 $${\mathrm{AODmax}} = 100 \cdot \frac{{\mathop {\sum}\nolimits_{i = 1}^{N_{{\mathrm{desat}}}} {{\mathrm{Smax}}_i} }}{{{\mathrm{TRT}}}},$$

34 $${\mathrm{AOD}}100 = 100 \cdot \frac{{\mathop {\sum}\nolimits_{i = 1}^{N_{{\mathrm{desat}}}} {{\mathrm{S}}100_i} }}{{{\mathrm{TRT}}}}.$$

Smax_*i*_ and S100_*i*_ are illustrated in Fig. 7.

#### Cumulative time (CT_*x*_)

**Fig. 8:**
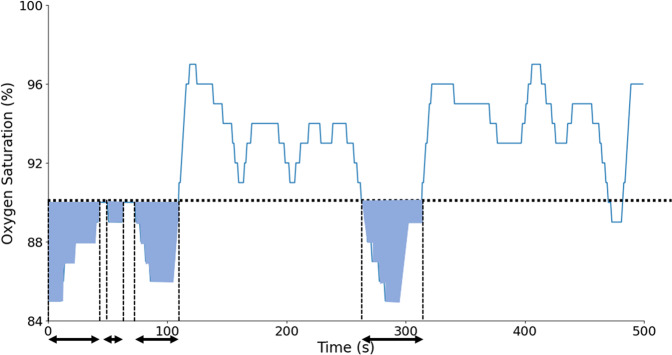
Illustration of CA90 and CT90 biomarkers. The sum of all the blue areas corresponds to CA90, whereas the sum of all the length arrows corresponds to CT90.

Percentage of the time spent below the *x*% oxygen saturation level. Typically, CT90 is used^55^ but other thresholds such as 80 or 84% have also been assessed^27^. This biomarker is evaluated on the overall signal, i.e., not only on the desaturation events, and it might consequently capture hypoxic behaviors that are different from the desaturation events found in OSA. The biomarker is illustrated in Fig. 8. It is mathematically defined as:

35 $${\mathrm{CT}}_x = 100 \cdot \frac{{\mathop {\sum}\nolimits_{i = 1}^{N_{{\mathrm{SpO}}2}} {t\left( x \right)_i} }}{{{\mathrm{TRT}} \ast {\mathrm{fs}}}},$$

36 $$t\left( x \right)_i = \left\{ {\begin{array}{ll} 1 & {\mathrm{if}}\,{\mathrm{SpO}}{2_i}\,<\,{x} \\ 0 & {\mathrm{else}} \end{array}}, \right.$$

where fs is the sampling frequency of the signal.

#### Cumulative area (CA_*x*_)

Total area under the *x*% oxygen saturation level. This biomarker was introduced by Watanabe et al.^54^, with *x* = 90%. Indeed, OSA patients tend to have a greater area under the baseline *x* than non-OSA patients and then get a higher value for this biomarker. The biomarker is illustrated in Fig. 8. It can be defined as:

37 $${\mathrm{CA}}_x = 100 \cdot \frac{{\mathop {\sum}\nolimits_{i = 1}^{N_{{\mathrm{SpO}}2}} {\left( {x - {\mathrm{SpO}}2\left( x \right)_i} \right)} }}{{{\mathrm{TRT}} \ast {\mathrm{fs}}}},$$

38 $${\mathrm{SpO}}2\left( x \right)_{\mathrm{i}} = \left\{ {\begin{array}{ll} {\mathrm{SpO}}2_i & {\mathrm{if}}\,{\mathrm{SpO}}2_i\, < \,{x} \\ x & {\mathrm{else}} \end{array}}. \right.$$

### Evaluation database

In order to demonstrate the usability of the implemented oximetry biomarkers and to define some normality ranges, we used the SHHS^39–42^ database. SHHS was a multi-center cohort study conducted by the National Heart Lung & Blood Institute (ClinicalTrials.gov Identifier: NCT0000527) to determine the cardiovascular and other consequences of sleep-disordered breathing. In all, 6441 men and women, aged ≥40 years, were enrolled between November 1, 1995 and January 31, 1998. Institutional review board from the Technion-IIT Rappaport Faculty of Medicine was obtained under number 62-2019 in order to use this database for research. The variable “ahi_a0h3a” was used for the AHI in order to define the classes. To elaborate this variable, the AHI was computed as the average number of all apneas and hypopneas (with oxygen desaturation >3% or an arousal) per hour of sleep and following the American Academy of Sleep Medicine (AASM) 2012 rules^56^. OSA severity was defined with respect to the AHI, i.e., mild (5 ≤ AHI < 15), moderate (15 ≤ AHI <30) or severe (AHI ≥ 30). The Nonin XPOD 3011 pulse oximeter (Nonin, USA) was used for recording. The signal was sampled at 1 Hz and with a resolution of ±0.01%. The OBM were computed for patients with available recordings and at least 4 h of continuous SpO_2_ tracing. This resulted in a total of 3806 individual patient recordings out of 5793 patients who participated in the first study visit (SHHS1). Among them, there were 1195 non-OSA, 1303 with mild OSA, 833 with moderate OSA, and 475 with severe OSA. OBMs were evaluated on these recordings in order to report reference ranges for each OBM.

### Statistical and regression analysis

The median and interquartile range of the SpO_2_ biomarkers were computed for the following classes: non-OSA, mild, moderate, and severe OSA. Kruskal–Wallis test with post hoc analysis was performed. Statistical significance or non-significance was indicated as “*p* < 0.05”, “*p* < 0.001”, or “*p* > 0.05”. Dunn post hoc analysis was performed between each pair of the classes. Multivariable linear regression was performed to assess the added value in combining OBM for the purpose of estimating the AHI. To this end, linear regression was performed between individual and combined sets of biomarkers and the AHI. For each model, the adjusted *R*^2^ $( {\overline R ^2} )$ score was reported. $\overline R ^2$ is defined as:

39 $$\overline R ^2 = 1 - \frac{{\left( {1 - R^2} \right)\left( {{\mathrm{size}} - 1} \right)}}{{{\mathrm{size}} - {\mathrm{pred}} - 1}},$$

where pred is the number of predictors, and size is the total sample size.

### Reporting summary

Further information on research design is available in the Nature Research Reporting Summary linked to this article.

## Supplementary information

Supplementary Information

Reporting Summary

## Data Availability

The SHHS database used in this research may be requested from sleepdata.org.

## Code availability

The source code used in this research is available at physiozoo.com.
