# Supplementary material for: Digital oximetry biomarkers for assessing respiratory function: standards of measurement, physiological interpretation, and clinical use
Source: NPJ Digit Med. 2021 Jan 4;4:1. doi: 10.1038/s41746-020-00373-5 (PMC7782845; doi:10.1038/s41746-020-00373-5)
Supplement: Supplementary file 1 — Supplementary Information [file 41746_2020_373_MOESM1_ESM.pdf]

Supplement

Supplementary Tables

Supplementary Table 1: Validation of the DFA, PSD, ApEn and PRSA functions. μ: mean; σ: standard deviation; RMSE: root mean square error; RMSE: normalized root mean square error. For DFA, the values estimated at each scale (4-64) were used for comparison.

	pobm		Benchmark			
	μ	σ	μ	σ	RMSE	NRMSE
DFA	3.42	3.38	3.35	3.26	0.22	0.06
PSD_total	93.62	325.23	93.58	325.26	6.35	0.06
PSD_band	5.63	7.26	5.48	7.05	0.23	0.04
PSD_ratio	0.07	6.52	0.06	6.89	3.5E-3	0.05
PSD_peak	0.06	3.24	0.06	3.24	1.2E-3	0.02
ApEn	0.39	0.09	0.39	0.09	3.5E-10	9.0E-10

Supplementary Table 2: Comparison of some biomarkers with results from other papers.

Name	This work	Published results
$DDmax_{\mu}$	4.93	4 [61]
AODmax	0.06	0.05 [28]
PODx	1.72	1.8 [28]
ΔI	0.36	0.14 [37]
CTMx	0.92	0.71 [37]
AV	95.90	96.00 [50]
Min	79.00	79.00 [50]
SD	1.42	2.40 [50]

Supplementary Table 3: Variables definition**.**

Symbol	Definition	Unit
N_{SpO2}	Number of samples of the SpO_2 time series	nu
n	Number of patients	nu
p	Number of nights recording	nu
f	Frequency of the signal	Hz
$SpO2_i$	Element i^{th} of the SpO_2 time series	%
N_{window}	Number of windows in the SpO_2 time series	nu
$SpO2\_window_i$	Average of the ith window of the SpO_2 time series	%
N_{desat}	Number of desaturations in the signal	nu
TRT	Total recording time.	sec
τ_i	Duration of the ith oxygen desaturation event.	sec
max_i	Maximum value of desaturation i	%
min_i	Minimum value of desaturation i	%
$Slope_i$	Slope of desaturation number i	$\%/sec$
$Smax_i$	Area of the specific desaturation event integrated from max	$\% * sec$
$S100_i$	Area of the specific desaturation event integrated from 100%	$\% * sec$
Δt_i	Time elapsed between desaturation i and desaturation $i - 1$	sec
NFFT	Number of points in the PSD signal	nu
r^2	Adjusted R-square score	nu

Supplementary Methods

Code quality control

The SHHS1 database was used to benchmark our implementations of DFA, PSD, ApEn and PRSA against established implementations of these functions. For that purpose, each recording of the SHHS1 was split into 1-hour window. The mean (μ) and the standard deviation (σ) for the evaluated functions were computed. The root means square error (RMSE) and normalized root means square error (NRMSE) between the mean of the pobm and benchmark functions were computed. The pobm DFA and PSD implementations were compared against the MATLAB DFA and PSD implementation available in PhysioZoo HRV. The ApEn function was compared against the ApEn implementation in MATLAB available from PhysioNet[1]. The PRSA window computation was benchmarked against the implementation of PRSA available in the PhysioNet cardiovascular signal toolbox[2]. For PRSA the RMSE between the windows was zero. For the other functions the results are reported in Table S2. The residual error for the DFA function may be due to the difference of languages: built-in functions for the line regression are used, Python in the case of the pobm toolbox and MATLAB in the case of the PhysioZoo toolbox. Similarly, for the PSD residual error where built-in functions for PSD estimation are used in different languages. For other OBM for which no reference open source benchmark code exists, we compared the estimated values of these biomarkers against their values reported in original research.

[1] URL: https://archive.physionet.org/physiotools/ApEn/
[2] URL: https://physionet.org/content/pcst/1.0.0/Tools/ECG_Analysis_Tools/

Algorithms pseudo-code:

$ApEn$**:**

1) Do the following for $m = M$ and $m = M + 1$:

   a. Divide the signal $x(n)$ into $N - m + 1$ vectors: $X[i] = [x(i), x(i+1), .., x(i+m-1)]$, where x is the SpO_2 signal to analyze.

   b. Define $d(X(i), X(j))$ as the maximum absolute difference between respective scalar components of the two vectors.

$$d\big(X(i), X(j)\big) = \max_{1 \le k \le m} |x(i+k-1) - x(j+k-1)|$$

   c. Compute $N^m(i), C^m(i)$ for each vector $X(i)$ defined as:

$$N^m(i) = \text{num}\{\, X(j) | d\big(X(i), X(j)\big) \le r\}$$

$$C^m(i) = {N^m(i)}\big/{(N - m + 1)}$$

   d. Compute $\varphi^m(r) = \frac{1}{N-m+1} \sum_{i=1}^{N-m+1} \ln(C^m(i))$

2) Compute $ApEn = \varphi^m(r) - \varphi^{m+1}(r)$

$SampEn(m, r)$:

1) Define $X_m(i) = \{x(i), \dots, x(i+m-1)\}$, where x is the SpO_2 signal to analyze.
2) Define $d[X_m(i), X_m(j)]$ as any distance function. In this case, the Euclidian function has been chosen.

3) Compute:

A: number of vector pair observing $d[X_{m+1}(i), X_{m+1}(j)] < r$

B: number of vector pair observing $d[X_m(i), X_m(j)] < r$

4) Return $-\ln(\frac{A}{B})$

DFA:

1) First integrate the signal:

$$y(k) = \sum_{i=0}^{k} x(i) - x_{avg}$$

where x_{avg} is the average of the signal, x is the SpO_2 signal to analyze.

2) Divide the obtained signal $y(k)$ into windows of length n. Let w be the number of windows obtained.

$$y_i = [y(i), y(i+1), .., y(i+n-1)], \qquad i = 0,1, .., w-1$$

3) For each window y_i, fit a least square line to the data, denoted $\overline{y_i}$, of size n too.

4) Compute

$$DFA(n) = \frac{1}{N}\sqrt{\sum_{i=0}^{w-1}\sum_{k=0}^{n-1}(y_i(k) - \overline{y_i}(k))^2}$$

$PRSA$:

1) Define anchor point $\mathrm{x(i)}$ as decreasing points in the signal: $\mathrm{x(i)} > \mathrm{x(i-1)}$. Note that decreasing points can also be used. Let M be the number of anchor points obtained.

2) For each anchor point $\mathrm{x_i}, \mathrm{i} = 1, .., \mathrm{M}$, define a window of length $2L$ around the point. Let $\mathrm{X_i}$ be the window around the anchor point x_i.

   Note that anchor points for which such a window cannot be computed are discarded. Furthermore, windows may overlap.

3) Average over the windows: $\bar{\mathrm{x}}(\mathrm{k}) = \frac{1}{\mathrm{M}} \sum_{\mathrm{i}=1}^{\mathrm{M}} \mathrm{X_i}[\mathrm{k}], \text{ for} - \mathrm{L} \leq \mathrm{k} < \mathrm{L}$. The result of the algorithm is a PRSA window of length 2L.

LZ:

1) Define the signal $P = \{s(1), s(2), \dots, s(n)\}$, where

$$s(i) = \begin{cases} 0 \; if \; SpO2_i < MED \\ 1 \; else \end{cases}$$

2) Let's denote SQ as the concatenation of sequences S and Q, $SQ\pi$ as the sequence SQ without the last character, $V(S)$ as the vocabulary of all different subsequences of S

3) Initialize $c(n) = 1, S = s(1), Q = s(2)$.

4) Until Q is the last character of P, do the following steps, starting with $r = 1$:

   a. Add $s(r + 2)$ to Q and judge if $Q \in V(SQ\pi)$.

   b. If not, increment r and return to previous step.

   c. Increment $c(n)$.

   d. Renew $S = \{s(1), s(2), \dots, s(r)\}$, $Q = s(r + 1)$.

5) Return $c(n)$.
