# Supplementary material for: Digital oximetry biomarkers for assessing respiratory function: standards of measurement, physiological interpretation, and clinical use
Source: NPJ Digit Med. 2021 Jan 4;4:1. doi: 10.1038/s41746-020-00373-5 (PMC7782845; doi:10.1038/s41746-020-00373-5)
Supplement: Supplementary file 2 — Reporting Summary [file 41746_2020_373_MOESM2_ESM.pdf]

nature research

Corresponding author(s): Joachim A. Behar

Last updated by author(s): 21-11-2020

Reporting Summary

Nature Research wishes to improve the reproducibility of the work that we publish. This form provides structure for consistency and transparency in reporting. For further information on Nature Research policies, see our Editorial Policies and the Editorial Policy Checklist.

Statistics

For all statistical analyses, confirm that the following items are present in the figure legend, table legend, main text, or Methods section.

n/a	Confirmed	
☐	☒	The exact sample size (*n*) for each experimental group/condition, given as a discrete number and unit of measurement
☒	☐	A statement on whether measurements were taken from distinct samples or whether the same sample was measured repeatedly
☐	☒	The statistical test(s) used AND whether they are one- or two-sided<br>*Only common tests should be described solely by name; describe more complex techniques in the Methods section.*
☒	☐	A description of all covariates tested
☐	☒	A description of any assumptions or corrections, such as tests of normality and adjustment for multiple comparisons
☐	☒	A full description of the statistical parameters including central tendency (e.g. means) or other basic estimates (e.g. regression coefficient) AND variation (e.g. standard deviation) or associated estimates of uncertainty (e.g. confidence intervals)
☐	☒	For null hypothesis testing, the test statistic (e.g. *F*, *t*, *r*) with confidence intervals, effect sizes, degrees of freedom and *P* value noted<br>*Give P values as exact values whenever suitable.*
☒	☐	For Bayesian analysis, information on the choice of priors and Markov chain Monte Carlo settings
☒	☐	For hierarchical and complex designs, identification of the appropriate level for tests and full reporting of outcomes
☐	☒	Estimates of effect sizes (e.g. Cohen's *d*, Pearson's *r*), indicating how they were calculated

Our web collection on statistics for biologists contains articles on many of the points above.

Software and code

Policy information about availability of computer code

Data collection	The Nonin XPOD 3011 pulse oximeter (Nonin, USA) was used for recording. The signal was sampled at 1Hz and with a resolution of ±0.01%.
Data analysis	The created software for analysis is made available at http://physiozoo.com/

For manuscripts utilizing custom algorithms or software that are central to the research but not yet described in published literature, software must be made available to editors and reviewers. We strongly encourage code deposition in a community repository (e.g. GitHub). See the Nature Research guidelines for submitting code & software for further information.

Data

Policy information about availability of data

All manuscripts must include a data availability statement. This statement should provide the following information, where applicable:

- Accession codes, unique identifiers, or web links for publicly available datasets
- A list of figures that have associated raw data
- A description of any restrictions on data availability

The SHHS database used in this research may be requested from sleepdata.org.

Field-specific reporting

Please select the one below that is the best fit for your research. If you are not sure, read the appropriate sections before making your selection.

☒ Life sciences ☐ Behavioural & social sciences ☐ Ecological, evolutionary & environmental sciences

For a reference copy of the document with all sections, see nature.com/documents/nr-reporting-summary-flat.pdf

Life sciences study design

All studies must disclose on these points even when the disclosure is negative.

Sample size	3806
Data exclusions	Recordings with available recordings (i.e. raw physiological waveforms) and at least 4 hours of continuous SpO2 tracing
Replication	Adjusted R^2
Randomization	n.a.
Blinding	n.a.

Reporting for specific materials, systems and methods

We require information from authors about some types of materials, experimental systems and methods used in many studies. Here, indicate whether each material, system or method listed is relevant to your study. If you are not sure if a list item applies to your research, read the appropriate section before selecting a response.

Materials & experimental systems

n/a	Involved in the study	
☒	☐	Antibodies
☒	☐	Eukaryotic cell lines
☒	☐	Palaeontology and archaeology
☒	☐	Animals and other organisms
☒	☐	Human research participants
☐	☒	Clinical data
☒	☐	Dual use research of concern

Methods

n/a	Involved in the study	
☒	☐	ChIP-seq
☒	☐	Flow cytometry
☒	☐	MRI-based neuroimaging

Clinical data

Policy information about clinical studies

All manuscripts should comply with the ICMJE guidelines for publication of clinical research and a completed CONSORT checklist must be included with all submissions.

Clinical trial registration	SHHS was a multi-center cohort study conducted by the National Heart Lung & Blood Institute (ClinicalTrials.gov Identifier: NCT0000527). Institutional review board from the Technion-IIT Rappaport Faculty of Medicine was obtained under number 62-2019 in order to use this database for research.
Study protocol	sleepdata.org
Data collection	sleepdata.org
Outcomes	Statistical significance or non-significance was indicated as “p<0.05”, “p<0.001" or “p>0.05”. Dunn post-hoc analysis was performed between each pair of the classes. Multivariable linear regression was performed to assess the added value in combining OBM for the purpose of estimating the AHI. To this end, linear regression was performed between individual and combined sets of biomarkers and the AHI. For each model, the adjusted R^2 〖(R ̅^2) score was reported.
